# Role of pelvic and para-aortic lymphadenectomy in abandoned radical hysterectomy in cervical cancer

**DOI:** 10.1186/s12957-016-1067-2

**Published:** 2017-01-14

**Authors:** Salim Abraham Barquet-Muñoz, Gabriel Jaime Rendón-Pereira, Denise Acuña-González, Monica Vanessa Heymann Peñate, Luis Alonso Herrera-Montalvo, Lenny Nadia Gallardo-Alvarado, David Francisco Cantú-de León, René Pareja

**Affiliations:** 1 Departamento de Ginecología Oncológica, Instituto Nacional de Cancerología (INCan), Av. San Fernando #22 Col. Sección XVI Del. Tlalpan, Ciudad de Mexico, CP 14080 Mexico; 2 Departamento de Ginecología Oncológica, Instituto de Cancerología de Las Américas (ICLA), Diagonal 75B No. 2A - 80/140, Medellin, Colombia; 3 Departamento de Gineconcologia, Hospital Medico Quirurgico y Oncologia, del Instituto Salvadoreño del Seguro Social, Alameda Juan Pablo II, San Salvador, El Salvador; 4 Dirección de Investigación, Instituto Nacional de Cancerología (INCan), Av. San Fernando #22 Col. Sección XVI Del. Tlalpan, Ciudad de Mexico, CP 14080 Mexico; 5 Subdirección de Investigación Clínica, Instituto Nacional de Cancerología (INCan), Av. San Fernando #22 Col. Sección XVI Del. Tlalpan, Ciudad de Mexico, CP 14080 Mexico; 6 Departamento de Ginecología Oncológica, Subdirección de Investigación Clínica, Instituto Nacional de Cancerología (INCan), Av. San Fernando #22 Col. Sección XVI Del. Tlalpan, Ciudad de Mexico, CP 14080 Mexico; 7 Clínica de Oncología ASTORGA, Calle 8 # 43 C 110, Medellin, Colombia

**Keywords:** Cervical cancer, Abandoned radical hysterectomy, Systematic lymphadenectomy

## Abstract

**Background:**

Cervical cancer (CC) occupies fourth place in cancer incidence and mortality worldwide in women, with 560,505 new cases and 284,923 deaths per year. Approximately, nine of every ten (87%) take place in developing countries. When a macroscopic nodal involvement is discovered during a radical hysterectomy (RH), there is controversy in the literature between resect macroscopic lymph node compromise or abandonment of the surgery and sending the patient for standard chemo-radiotherapy treatment. The objective of this study is to compare the prognosis of patients with CC whom RH was abandoned and bilateral pelvic lymphadenectomy and para-aortic lymphadenectomy was performed with that of patients who were only biopsied or with removal of a suspicious lymph node, treated with concomitant radiotherapy/chemotherapy in the standard manner.

**Methods:**

A descriptive and retrospective study was conducted in two institutions from Mexico and Colombia. Clinical records of patients with early-stage CC programmed for RH with an intraoperative finding of pelvic lymph, para-aortic nodes, or any extracervical involvement that contraindicates the continuation of surgery were obtained. Between January 2007 and December 2012, 42 clinical patients complied with study inclusion criteria and were selected for analysis.

**Results:**

In patients with CC whom RH was abandoned due to lymph node affectation, there is no difference in overall survival or in disease-free period between systematic lymphadenectomy and tumor removal or lymph node biopsy, in pelvic lymph nodes as well as in para-aortic lymph nodes, when these patients receive adjuvant treatment with concomitant radiotherapy/chemotherapy.

**Conclusions:**

This is a hypothesis-generator study; thus, the recommendation is made to conduct randomized prospective studies to procure better knowledge on the impact of bilateral pelvic and para-aortic lymphadenectomy on this group of patients.

## Background

Cervical cancer (CC) occupies fourth place in cancer incidence and mortality worldwide in women, with 560,505 new cases and 284,923 deaths per year. Approximately, nine of every ten (87%) of the latter take place in developing countries [1]. In 2009, the International Federation of Gynecology and Obstetrics (FIGO) redefined CC staging, reiterating that this must be clinical, to be more accessible to sectors and countries with low resources [2]. However, it must be considered that clinical staging entails a high percentage of error, particularly in knowledge of the pelvic lymph node and para-aortic involvement [3]. The National Comprehensive Cancer Network (NCCN) recommends radical hysterectomy (RH) with bilateral pelvic lymphadenectomy (BPL) for patients with CC in early stages (IA2–IB1) and who do not wish to preserve fertility; in the case of finding lymph node or extracervical spread during the surgical approach, the RH should be suspended, in order to carry out a pathological examination of the para-aortic lymph nodes and to administer treatment with concomitant radiotherapy (RT) and chemotherapy (ChemT) (RT/ChemT) [4]. The Gynecologic Oncology Group (GOG) in 2000 published a study where the main reason for suspending a RH in patients with early stage CC was the presence of lymph node involvement [5]. At present, studies have not demonstrated the benefit of completing RH in the case of trans-surgical findings of extra-cervix tumor spread [6, 7]. However, para-aortic lymphadenectomy (PAL) has the intent of surgical staging to establish the need for extending radiation fields, although controversy continues to exist concerning its usefulness in prognosis [8]. In addition, there is controversy in the literature between resect macroscopic lymph node compromise or abandonment of the surgery and sending the patient for standard radical treatment.

The objective of this study is to compare the prognosis of patients with CC whom RH was abandoned and BPL and PAL was performed, with that of patients who were only biopsied or with removal of a suspicious lymph node, treated with concomitant RT/ChemT in the standard manner.

## Methods

A descriptive and retrospective study was conducted in two institutions from Mexico and Colombia. Clinical records of patients with early stage CC programmed for RH with an intraoperative finding of pelvic lymph, para-aortic nodes, or any extracervical involvement that contraindicates the continuation of surgery were obtained. Patients with non-invasive CC, with any other primary tumor, who were submitted to surgery after RT and/or ChemT, or who had incomplete clinical records were excluded from the study. The project was approved by the Ethics and Research Committees of the Instituto Nacional de Cancerología (INCan) of Mexico and Instituto de Cancerología de Las Américas (ICLA), Medellin, Colombia.

The following variables were evaluated: country, age, body mass index (BMI), histology, clinical stage, size of the pathological tumor, presence of lymphovascular space involvement (LVSI), histological grade, reason for abandonment of surgery, type of lymphadenectomy or tumor removal, surgical time, trans-surgical bleeding, transfusion, presence of other complications related with the surgical procedure, duration of definitive post-surgical treatment, and type of recurrences.

BPL was defined as bilateral resection of all lymph nodes between the circumflex vein as inferior margin, 2 cm above the external iliac-artery bifurcation as superior margin, the genitocrural nerve as lateral margin, the superior vesical artery as medial margin, and the obturator nerve as dissection floor. With regard to PAL, lateral margins were the ureters, the inferior margin was 2 cm above the external iliac-artery bifurcation, and the superior margin at least to the inferior mesenteric artery. Tumor removal was defined as resection of solely the macroscopic lymph node disease, and lymph node biopsy (LNB), as taking a portion of the macroscopically affected lymph node. In data analysis, surgical procedures were not mutually exclusive. Surgical time was defined as from skin incision to closing of the latter. Complications related with the procedure were those that presented during 7 days after the surgical procedure.

After the procedure, all of the patients received definitive treatment. Patients who had pelvic lymph node affectation exclusively received concomitant ChemT/RT and brachytherapy (BT) or external RT with BT exclusively. In the case of para-aortic disease, extended fields of the para-aortic region were included according to guidelines established for this procedure. If there was a disease in other organs, the patients received platinum-based chemotherapy. Treatment duration was defined as time between initiation of definitive oncologic treatment and finalization of the latter. Disease-free period (DFP) in months was defined as time between finalization of definitive treatment and presence of recurrence, the patient’s last visit, or last follow-up. Overall survival (OS) in months was defined as the period between diagnosis and the patient’s death or last follow-up visit.

Descriptive statistics was carried out for demographic variables, reporting central tendency medians. Univariate analysis was performed by Mann–Whitney *U* test for continuous variables, and the chi-squared or the Fisher exact test according to the case for ordinal variables. In the case of its being feasible, the variables were included in the multivariate Cox analysis. OS and DFP were analyzed by the Kaplan–Meier method, and curves were compared utilizing the log-rank test. Statistical significance was defined with a value of *p* <0.05. The IBM SPSS statistical package for Mac (2013) was used.

## Results

Between January 2007 and December 2012, 42 clinical patients complied with study inclusion criteria and were selected for analysis. Clinicopathological characteristics are described in Table 1. Median age was 46 years (interquartile range (IQR), 40.8–56), with a BMI of 25.7 (IQR, 23.2–29.5). Twenty (42.6%) patients were submitted to some imaging study; of these, 10 (50%) had computed tomography (CT), being the most frequently utilized study for evaluation. The most common histology was squamous cell in 24 (57.1%) cases, followed by adenocarcinoma in 12 (28.6%), adenosquamous in 4 (9.5%), and other histologies in 2 cases (4.8%). In 37 (88.1%) cases, initial clinical stage was IB1, with 2 (4.8%) in IB2 and with 3 (7.1%) in IIA1. Median tumor size was 3.0 cm (IQR, 2.0–4.0). Fourteen (33.3%) cases were well-differentiated, 17 (40.1%) moderately differentiated, and 11 (26.2%) poorly differentiated. Only 5 (12%) patients presented lymphovascular involvement.

**Table 1:** Clinical and pathological characteristic of patients with cervical cancer with abandoned hysterectomy (*N* = 42)

Country^a^	
Mexico (INCan)	19 (45)
Colombia (ICLA)	23 (55)
Age (years)^b^	46 (40.8–56)
BMI^b^	25.68 (23.2–29.5)
Prior imaging study^a^	20 (42.6)
Ultrasonography	7 (35)
CT	10 (50)
MRI	3 (15)
Histology^a^	
Squamous	24 (57.1)
Adenocarcinoma	12 (28.6)
Adenosquamous	4 (9.5)
Others	2 (4.8)
Clinical stage^a^	
IB1	37 (88.1)
IB2	2 (4.8)
IIA1	3 (7.1)
Tumor size (cm)^b^	3.0 (2.0–4.0)
Tumor grade^a^	
Well-differentiated	14 (33.3)
Moderately differentiated	17 (40.1)
Poorly differentiated	11 (26.2)
LVSI^a^	5 (12)
Reason for abandonment^a^	
Pelvic lymph node affectation	19 (45.2)
Para-aortic lymph node affectation	9 (21.4)
Parametrial involvement	7 (16.7)
Others	7 (16.7)
Type of lymphadenectomy^a^	
Pelvic	14 (33.3)
Para-aortic	6 (14.3)
Tumor biopsy	
Pelvic	19 (45.2)
Para-aortic	14 (33.3)

*BMI* body mass index, *CT* computed tomography, *MRI* magnetic resonance imaging, *LVSI* lymphovascular space involvement

^a^ *N* (relative frequency)

^b^Median (interquartile range)

The principal reason for not performing the RH was the presence of lymph node disease in 28 (66.6%) patients, followed by parametrial involvement in 7 (16.7%) patients, and due to other reasons in 7 (16.7%) patients. Among patients in whom the reason was lymph node involvement, pelvic lymph node disease was present in 19 (67.8%) cases, followed by para-aortic lymph nodes in 9 (32.2%) cases. Thirty-three (78.6%) patients were submitted to some type of surgical procedure on the pelvic lymph nodes, and 20 (47.6%) patients had some type of procedure on para-aortic lymph nodes. These procedures were not mutually exclusive.

Of the 33 patients who had undergone some surgical procedure in pelvic lymph nodes, 19 (57.6%) patients underwent tumor removal or LNB and 14 (42.4%) underwent BPL (Table 2). Median number of lymph nodes obtained in the BPL group was 14 (IQR, 8–21). A statistically significant difference was found in terms of the institution of origin (*p* = 0.02), with a distribution in the case of tumor removal or LNB of 10 (52.6%) patients for INCan and 9 (47.4%) for ICLA, while in the case of BPL, distribution was 2 (14.3%) and 12 (85.7%). A difference was also found with respect to BMI (*p* = 0.01), with 27.4 (IQR, 25.3–31.1) for patients who underwent tumor removal or LNB vs. 24.6 (IQR, 22.4–25.4) for patients submitted to BPL. Statistically significant differences were not found in terms of age, histology, clinical stage, tumor size, grade of tumor, presence of lymphovascular space involvement, surgical time, amount of bleeding, transfusions, other types of complications, hospital stay, duration of definitive treatment, or presence of recurrences. Two surgically related complications were observed: one hydroelectrolytic decompensation in the BPL group and one pulmonary thromboembolism in the tumor removal or LNB group, without this achieving a statistically significant difference (*p* = 0.82).

**Table 2:** Comparison of patients group with pelvic lymph node surgery

	Tumor removal and/or pelvic biopsy *N* = 19 (57.6%)	Pelvic lymphadenectomy *N* = 14 (42.4%)	*p*
Country^a^			
Mexico (INCan)	10 (52.6)	2 (14.3)	0.02
Colombia (ICLA)	9 (47.4)	12 (85.7)	
Age (years)^b^	45 (35-53)	45.5 (40-54.3)	0.55
BMI ^b^	27.4 (25.3–31.1)	24.6 (22.4–25.4)	0.01
Histology^a^			
Squamous	9 (47.4)	9 (64.3)	0.22
Adenocarcinoma	5 (26.3)	5 (35.7)	
Adenosquamous	3 (15.8)	0	
Others	2 (10.5)	0	
Initial clinical stage^a^			
IB1	18 (94.7)	13 (92.9)	0.82
IB2	1 (5.3)	1 (7.1)	
Tumor size (cm)^b^	3 (1.6–4)	3 (2–4)	0.63
Tumor grade^a^			
Well-differentiated	4 (21.1)	7 (50)	0.05
Moderately differentiated	7 (36.8)	6 (42.9)	
Poorly differentiated	8 (42.1)	1 (7.1)	
LVSI^a^	3 (15.8)	2 (14.3)	0.74
Systematic PAL^a^	1 (5.3)	5 (35.7)	0.062
BPR^a^	11 (57.9)	3 (21.4)	0.073
PR positive	17 (89.5)	12 (85.7)	NS
Surgical time (min)^b^	150 (120–190)	165 (103.8–198.8)	0.69
Blood loss (mm)^b^	100 (70–250)	100 (57.5–212.5)	0.78
Blood transfusion^a^	1 (0.05)	1 (0.07)	0.82
Others complications^a^	1 (0.05)	1 (0.07)	0.82
Hospital stay (days)^b^	2 (2–2)	2 (2–3.3)	0.19
Neoadjuvant treatment term (weeks)^b^	10 (7.5–14)	8.8 (8.1–11)	0.57
Recurrence^a^	7 (26.8)	2 (14.3)	0.15
Local	1 (14.3)	0	
Regional	6 (85.7)	2 (100)	
Distance	0	0	

Confidence interval 95%

*BMI* body mass index, *Mod* moderate, *LVSI* lymphovascular space involvement, *PAL* para-aortic lymphadenectomy, *BPR* biopsy or pelvic lymph node removal, *PR* pathology report, *NS* not significant

^a^ *N* (relative frequency)

^b^Median (interquartile range)

Of patients on whom some para-aortic lymph node procedure was performed, 14 (70%) had tumor removal or LNB and 6 (30%) had PAL (Table 3). Median number of lymph nodes obtained in the PAL procedure was 6 (IQR, 4–6). In the group of tumor removal or LNB, 6 (42.9%) cases had squamous histology and 5 (35.7%) of adenocarcinoma, while in the PAL group, there were 3 (50%) cases of squamous and 3 (50%) of adenocarcinoma (*p* = 0.021). Likewise, there was a difference (*p* < 0.001) with respect to stages: in the tumor removal group, there were 12 (85.7%) in stage IB1 and 2 (14.3%) in IB2, while in the PAL group, there were 6 (100%) in IB1. In terms of recurrence in para-aortic procedure group, 3 (21.4%) regional and 3 (21.4%) distant metastasis were found in the tumor removal or the para-aortic LNB group, and only 1 (16.7%) local recurrence was observed in the PAL group (*p* = 0.04). Regarding complications, only 1 (16.7%) patient presented hydroelectrolytic decompensation, who was the same as reported in the comparison with BPL. With regard to the pathology report, in the group of LNB or tumor removal, 6 (42.9%) patients had lymph node metastasis, and in the PAL group, there was 1 (16.7%) without a statistically significant difference (*p* = 0.354). No statistically significant difference was observed in the remainder of the variables analyzed.

**Table 3:** Comparison of patients group with para-aortic lymph node surgery

	Tumor removal and/or para-aortic biopsy *N* = 14 (70.0%)	Para-aortic lymphadenectomy *N* = 6 (30.0%)	*p*
Country^a^			
Mexico (INCan)	5 (35.7)	2 (33.3)	0.26
Colombia (ICLA)	9 (64.3)	4 (66.7)	
Age (years)^b^	45 (40–45)	44.5 (42–50)	0.90
BMI^b^	26.55 (23.7–28.3)	23.9 (22.5–32.7)	0.60
Histology^a^			
Squamous	6 (42.9)	3 (50)	0.02
Adenocarcinoma	5 (35.7)	3 (50)	
Adenosquamous	1 (7.1)	0	
Others	2 (14.3)	0	
Initial clinical stage^a^			
IB1	12 (85.7)	6 (100)	<0.001
IB2	2 (14.3)	0	
Tumor size (cm)^b^	3.5 (2–4)	3.15 (2.9–4)	
Tumor grade^a^			
Well-differentiated	4 (28.6)	3 (50)	0.95
Moderately differentiated	3 (21.4)	3 (50)	
Poorly differentiated	7 (0.5)	0	
LVSI^a^	3 (21.4)	0	0.09
PR positive	6 (42.9)	1 (16.7)	0.354
Surgical time (min)^b^	172.5 (90–195)	120 (100–195)	0.78
Blood loss (mm)^b^	100 (70–300)	140 (100–250)	0.72
Blood transfusion^a^	0	0	NS
Others complications^a^	0	1 (16.7)	<0.001
Hospital stay (days)^b^	2 (2–2)	2 (2–3)	0.44
Neoadjuvant treatment term (weeks)^b^	10.5 (8.2–14.2)	10.5 (8–15.4)	0.66
Recurrence^a^			
Local	0	1 (16.7)	<0.001
Regional	3 (21.4)	0	
Distance	3 (21.4)	0	

Confidence interval 95%

*BMI* body mass index, *Mod* moderate, *LVSI* lymphovascular space involvement, *PR* pathology report

^a^ *N* (relative frequency)

^b^Median (interquartile range)

**Fig. 1:**
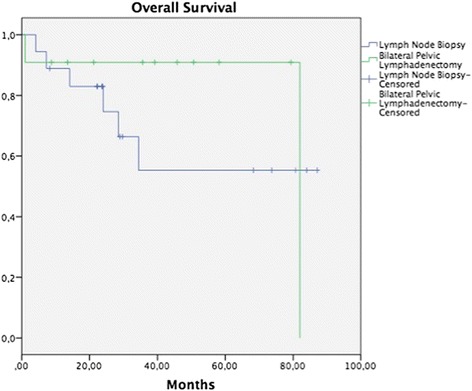
Overall survival (OS) in patients with pelvic lymph node procedure. Five-year OS in the LNB group was 55.5%, and in the BPL group 90.9% (*p* = 0.171)

**Fig. 2:**
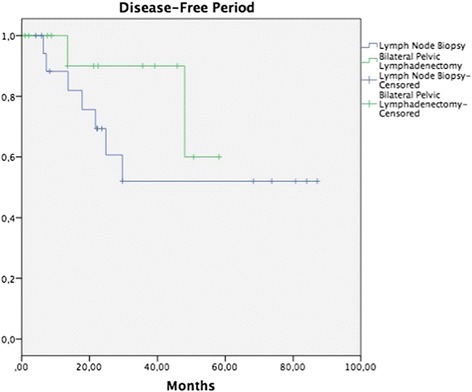
Disease-free survival (DFS) in patients with pelvic lymph node procedure. Five-year DFS in the LNB group was 52%, and in the BPL group 60% (*p* = 0.265)

**Fig. 3:**
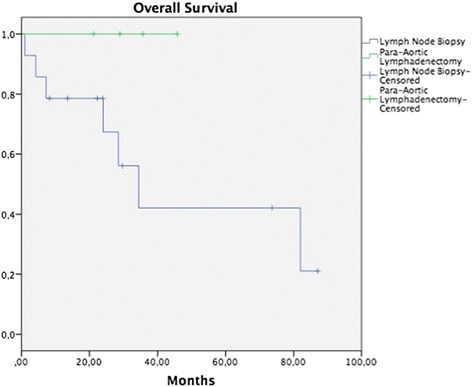
Overall survival (OS) in patients with para-aortic lymph node procedure. Five-year OS in the LNB group was 42.1%, and in the PAL group 100% (*p* = 0.126)

**Fig. 4:**
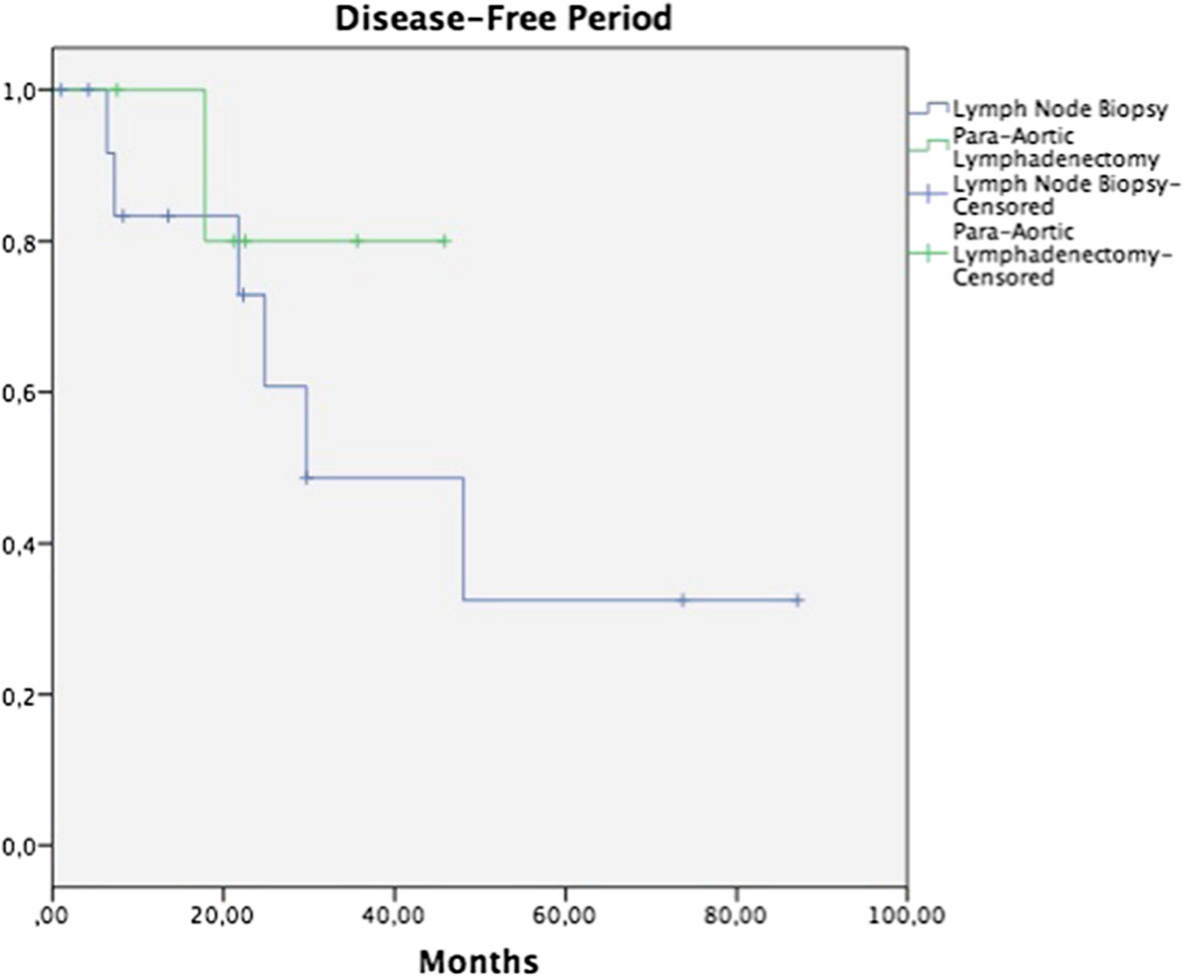
Disease-free survival (DFS) in patients with para-aortic lymph node procedure. Five-year DFS in the LNB group was 32.5%, and in the PAL group 80% (*p* = 0.437)

Median follow-up was 32.2 months (range, 5.7–93.8 months). In patients on whom some surgical procedure involving pelvic lymph nodes had been performed, 5-year OS in the tumor removal or LNB group was 55.5 vs. 90.9% in the group with BPL, without a statistically significant difference (*p* = 0.171) (Fig. 1), while DFP was 52.0 vs. 60%, respectively, without a statistically significant difference (*p* = 0.265) (Fig. 2). In patients on whom a surgical procedure was performed on para-aortic lymph nodes, 5-year OS in the tumor removal or the LNB group was 42.1%, and 100% in the PAL group, without a significant difference (*p* = 0.126) (Fig. 3), while 5-year DFP was 32.5 and 80%, respectively, without a statistically significant difference (*p* = 0.437) (Fig. 4).

## Discussion

In early stages of CC, the probability of pelvic lymph node metastasis is 2 to 8%, while para-aortic involvement is extremely rare [9]. The most important prognostic factor in these patients is lymph node disease: in stages IB or IIA with negative pelvic lymph nodes, 5-year OS is 88–96%, compared with 50–74% at similar stages with metastasis to lymph nodes [10]. The results are worse if there is para-aortic disease [11]. The main cause for abandonment of RH is the trans-surgical presence of affected lymph nodes. The GOG in the year 2000 analyzed patients with stage IB1 CC on whom HR was abandoned. Of 1127 patients, 68 (8.7%) underwent abandonment of their surgical procedure, finding that the majority of these were due to lymph node metastasis: in 25 patients by para-aortics and in 12 by pelvic nodes [5]. In our study, the main cause for abandonment of RH was the presence of lymph node disease in 66.6%. The majority was due to pelvic lymph nodes in 19 cases, in comparison with 9 cases with para-aortic metastatic disease. It is important to consider that only 42.6% of patients had at least one imaging study prior to the surgical procedure, with CT being the most frequently utilized imaging study, and that in none of these patients was there suspicion of tumor activity at the lymph node level (Table 1). This is probably because, in early CC stages, management guidelines do not recommend the performance of routine imaging studies [4]. One must also consider that the sensitivity and specificity of CT in diagnosing lymph node disease ranges from 64.7 to 96.6% [12]. Finally, it is probable that patients with results of lymph node suspicion were never programmed for a surgical procedure, but were instead sent to treatment with concomitant RT/ChemT.

In the literature, there are studies that do not demonstrate a benefit on completing RH in the case of finding trans-surgical lymph node metastasis. In 2008, Richard et al. compared two groups of patients obtained from the Surveillance Epidemiology and End Results (SEER) study with a diagnosis of stage IB1 CC and lymph node metastasis; patients in the former group were submitted to RH with BPL and PAL, and in patients in the latter group, RH was aborted and only BPL and PAL were carried out, both groups receiving adjuvant RT. With a follow-up of more than 6 years, a difference was not found in 5-year OS (69 vs. 71%; *p* = 0.45) and in death due to disease (31 vs. 24%; *p* = 0.4), the treatment was considered equivalent in both groups [6]. Gray et al. analyzed 268 women with CC with early stages, RH was not completed in 7%; among these, the main reason was macroscopic pelvic lymph node disease (84%), and in the remainder other causes of extracervical disease. Of the group in which RH was aborted, 17% recurred vs. 8% of patients on whom the surgery was completed (*p* = 0.168). Likewise, a difference was not found in OS in the group with abandoned RH vs. those on whom RH was performed (73 vs. 80%; *p* = 0.772). A difference was also not found in the rate of surgical complications between both groups (10 vs. 11%; OR, 0.92; 95% CI, 0.08–6.633) [7]. However, Suprasert et al. found a difference in the prognosis between to continue or to abandon the surgical procedure. These authors found that although there was no significant difference in the rate of complications of both groups, a superior 2-year DFP was found in patients submitted to RH in comparison with those on whom the procedure was abandoned (93.5 vs. 58.5%; *p* = 0.01). On the other hand, there were differences in the amount of positive lymph nodes and in the amount of patients who received extended-field RT [13].

A search was conducted in the literature that analyzes the prognosis of carrying out systematic BPL in patients with abandoned RH due to trans-surgical findings; however, no publication was identified on this theme. In our study, the performance of BPL vs. tumor removal or LNB was compared, without finding a difference in OS and DFP between the groups (Figs. 1, 2, 3, and 4). It would appear that treatment with post-surgical RT or concomitant ChemT/RT is sufficient without performing a more extensive surgical procedure. In addition, it is noteworthy that median BMI was higher in patients of the tumor removal or LNB group in comparison with those of the BPL group (27.4 vs. 24.6; *p* = 0.01) and that this could be why there are fewer patients on whom a more extensive surgical procedure was performed. Similarly, it appears that at INCan (14%), a lesser number of extensive procedures was performed in comparison with ICLA (86%); however, this type of decision can be influenced by the higher BMI in the tumor removal or LNB group. In terms of surgical time, bleeding, or other complications, need for transfusions, hospital stay, and treatment duration, there is no statistically significant difference between the two groups. Although we would expect that a difference would exist clinically, absence of the latter can be explained in part by our study’s sample size.

In our group of patients, there was no difference between OS and DFP in patients on whom PAL was performed vs. tumor removal or LNB. This can be due to that all patients received extended-field RT. In the study of Varia and collaborators, 95 women with CC and para-aortic affectation were treated with concomitant ChemT/RT (extended-field), finding a 3-year OS and a 3-year DFP of 39 and 34%, respectively, and grade 3 or greater toxicity rate of 19%, suggesting that control of the disease can be achieved with this modality [14]. In our series of patients on whom PAL was carried out, no patients presented lymph node recurrence, but these patients did present a higher rate of trans-surgical complications, in contrast with those only submitted to tumor removal or LNB. These results must be regarded with a certain reservation due to the limited number of cases; however, this did suggest that patients not submitted to PAL can present microscopic tumor activity and that this can be the cause of para-aortic recurrence. The role of PAL in patients with lymph node affliction remains controversial. In the study of Lai and coworkers, the authors analyzed the advantage of performing PAL on patients with CC in comparison with carrying out imaging studies, finding longer OS (*p* = 0.024) and shorter DFP (*p* = 0.003) in the group of surgical patients [15]. In the study of Chantalat et al., a difference in OS was not found between patients who had been submitted to PAL and those who not (*p* = 0.31) [16].

To our knowledge, this is the first study to evaluate the role of BPL, in particular in patients whose HR surgical procedure was abandoned because of suspicion of lymph node affectation. These results should be taken with reserve. Among the study’s limitations, we find its retrospective characteristics, the short follow-up, the upper limit of PAL is not known, and few patients have been evaluated, in addition to that the ChemT or RT type of definitive treatment and its duration or initiation can differ between the two participating institutions.

## Conclusions

In patients with CC on whom RH was abandoned due to lymph node affectation, there is no difference in OS or in DFP between systematic lymphadenectomy vs. tumor removal or LNB, in pelvic lymph nodes as well as in para-aortic lymph nodes, when these patients receive adjuvant treatment with concomitant RT/ChemT. This is a hypothesis-generator study; thus, the recommendation is made to conduct randomized prospective studies to procure better knowledge on the impact of BPL and PAL on this group of patients.

## Abbreviations

BMI: Body mass index
BPL: Bilateral pelvic lymphadenectomy
BT: Brachytherapy
CC: Cervical cancer
ChemT: Chemotherapy
CT: Computed tomography
DFP: Disease-free period
FIGO: International Federation of Gynecology and Obstetrics
GOG: Gynecologic Oncology Group
ICLA: Instituto de Cancerología de Las Américas
INCan: Instituto Nacional de Cancerologia
IQR: Interquartile range
LNB: Lymph node biopsy
LVSI: Lymphovascular space involvement
NCCN: The National Comprehensive Cancer Network
OS: Overall survival
PAL: Para-aortic lymphadenectomy
RH: Radical hysterectomy
RT: Radiotherapy
